# Preserved search asymmetry in the detection of fearful faces among neutral faces in individuals with Williams syndrome revealed by measurement of both manual responses and eye tracking

**DOI:** 10.1186/s11689-017-9190-0

**Published:** 2017-03-03

**Authors:** Masahiro Hirai, Yukako Muramatsu, Seiji Mizuno, Naoko Kurahashi, Hirokazu Kurahashi, Miho Nakamura

**Affiliations:** 1 Institute for Developmental Research, Aichi Human Service Center, 713-8 Kagiya-cho, Kasugai, Aichi 480-0392 Japan; 2 Department of Pediatrics, Central Hospital, Aichi Human Service Center, 713-8 Kagiya-cho, Kasugai, Aichi 480-0392 Japan; 3 Present Address: Center for Development of Advanced Medical Technology, Jichi Medical University, 3311-1 Yakushiji, Shimotsuke, Tochigi 392-0498 Japan

**Keywords:** Williams syndrome, Visual search, Fearful face detection, Search asymmetry, Attention, Eye tracking

## Abstract

**Background:**

Individuals with Williams syndrome (WS) exhibit an atypical social phenotype termed hypersociability. One theory accounting for hypersociability presumes an atypical function of the amygdala, which processes fear-related information. However, evidence is lacking regarding the detection mechanisms of fearful faces for individuals with WS. Here, we introduce a visual search paradigm to elucidate the mechanisms for detecting fearful faces by evaluating the search asymmetry; the reaction time when both the target and distractors were swapped was asymmetrical.

**Methods:**

Eye movements reflect subtle atypical attentional properties, whereas, manual responses are unable to capture atypical attentional profiles toward faces in individuals with WS. Therefore, we measured both eye movements and manual responses of individuals with WS and typically developed children and adults in visual searching for a fearful face among neutral faces or a neutral face among fearful faces. Two task measures, namely reaction time and performance accuracy, were analyzed for each stimulus as well as gaze behavior and the initial fixation onset latency.

**Results:**

Overall, reaction times in the WS group and the mentally age-matched control group were significantly longer than those in the chronologically age-matched group. We observed a search asymmetry effect in all groups: when a neutral target facial expression was presented among fearful faces, the reaction times were significantly prolonged in comparison with when a fearful target facial expression was displayed among neutral distractor faces. Furthermore, the first fixation onset latency of eye movement toward a target facial expression showed a similar tendency for manual responses.

**Conclusions:**

Although overall responses in detecting fearful faces for individuals with WS are slower than those for control groups, search asymmetry was observed. Therefore, cognitive mechanisms underlying the detection of fearful faces seem to be typical in individuals with WS. This finding is discussed with reference to the amygdala account explaining hypersociability in individuals with WS.

## Background

Williams syndrome (WS) is a rare genetic disorder caused by the deletion of approximately 28 genes in chromosome 7 [1, 2]. The prevalence of WS ranges from 1 in 7500 to 1 in 20,000 [1, 2]. Besides the physical characteristics associated with WS, such as dysmorphic facial features and heart defects, a unique cognitive and behavioral profile has been described. Behaviorally, WS is characterized by hypersociability [3], which is characterized by interest in both familiar and unfamiliar people [3–8].

A previous theoretical framework has proposed two accounts for hypersociability, namely the frontal lobe account and the amygdala account [8–10]. The frontal lobe of the brain is associated with cognitive processes, such as attention setting and shifting, working memory, and planning, that underlie goal-directed behavior [11]. The frontal lobe account of hypersociability postulates that atypical social interest in other people may be caused by the impaired inhibition of the desire to approach both familiar and unfamiliar people due to an atypical brain structure and function in the frontal lobe [8]. Recent neuroimaging studies have revealed that individuals with WS show atypical increases in gray matter volumes in the frontal lobes [12]. Another finding showed difficulties in executive functions similar to those seen in individuals with attention deficit and hyperactivity disorders (ADHD) [13]. Further evidence such as a study employing a rating system for approachability implies that the atypical social approachability of individuals with WS is not due to difficulties in the recognition of emotion but due to the problem of inhibition [7, 8]. Other neuroimaging evidence has suggested that the dorsolateral frontal cortex and dorsal anterior cingulate cortices in the WS group were significantly reduced in neural activity compared with typical controls during a Go/No-go inhibition task [14].

The alternative amygdala account postulates that atypical social approachability may be due to the atypical structure and neural activity of the amygdala. The amygdala is a part of the limbic system controlling socioemotional behavior, considered to process fear-related information [15]. A previous behavioral study has reported that individuals with WS displayed atypical positive social judgment of unfamiliar faces showing both positive and negative emotions [16]. Recent neuroimaging studies have shown that a positive relationship between the right amygdala volume and approachability ratings, particularly ratings of “negative” faces [17], was found in individuals with WS. Moreover, individuals with WS showed atypical amygdala response to fearful expressions [18]. Furthermore, when individuals with WS observed both fearful faces and fearful scenes, neural activity in the amygdala and middle prefrontal cortex showed a contrast with the activity of the age- and gender-matched controls [19]. For the age- and gender-matched control group, the neural activity of the amygdala in response to fearful faces was significantly enhanced compared to that in response to fearful scenes. In contrast to the neural activity occurring in controls, the neural response of the amygdala in WS individuals in response to fearful scenes was enhanced compared with that in response to fearful faces.

However, it remains unclear whether the amygdala account can fully explain hypersocial behavior in individuals with WS. For example, according to a neuropsychological study, the patient S.M., who did not have WS, showed complete bilateral amygdala destruction since late childhood as a consequence of Urbach–Wiethe disease. She exhibited preserved behavioral performance in terms of the *detection* of fearful faces [20]. Additionally, findings from prefrontal theory imply that the performance of the *recognition* of fearful faces was preserved in individuals with WS (e.g., [7]). This evidence motivated us to test whether the performances of the *detection* of fearful faces were preserved in individuals with WS.

One way of characterizing the strategy for detecting a fearful face is to adopt a visual search paradigm and evaluate visual search asymmetry as an index. Visual search asymmetry is defined as the case in which the reaction time of searching for target stimulus *A* among distractor stimuli *B* is more prolonged than that of searching for target stimulus *B* among distractor stimuli *A* [21–23]. Targets defined by the presence of a basic preattentive feature (e.g., stimulus *A*) are more easily found in a search than among distractors lacking such a feature (e.g., stimuli *B*) compared with the case in which the search and distractors were swapped [22]. Search asymmetries can be observed in the presence and absence of low-level features, such as color, orientation, and motion information [21, 23], as well as in higher levels of visual features, such as the direction of the gaze [24, 25], identification of letters, [26], figures [23], and biological motion [27]. Regarding emotional faces, previous studies have demonstrated that fearful faces were more quickly detected than happy or neutral faces by both children and adults [28] as well as infants [29]. Further, search asymmetry has also been reported between emotional and neutral faces [e.g., [30]).

Atypical cognitive and neural mechanisms underlying the perception of faces in individuals with WS have been reported [31–33]. Face perception is well known to require configural processing, integrating several parts into a coherent figure. Studies have reported atypical visuospatial processing in people with WS [34–36]. To investigate the configural processing of faces, an inversion paradigm has been used, where an upside-down face is presented to participants. Inverting a face is thought to disrupt the configural processing of the face [37, 38] but not the processing of low-level image-based properties. The face inversion effect is defined as a greater decrease in recognition performance for faces than for other mono-oriented objects presented upside down [39–41]. Although several studies on face perception and recognition have shown that individuals with WS exhibit normal performance accuracy on face discrimination tasks, including the recognition of unfamiliar upright faces [32, 42, 43], the evidence of them exhibiting an inversion effect is not strong [31–33]. Furthermore, studies using behavioral response [44], electroencephalography (EEG) [45], and magnetoencephalography (MEG) [46] have shown that some aspects of facial processing may be delayed or atypical in individuals with WS. These cumulative findings suggest that facial processing in individuals with WS is atypical. However, whether and how the processing of emotional faces, particularly of fearful faces, is modulated by this remains unclear.

In previous studies [47, 48], we demonstrated that attentional capture by the presence of an upright face that is not a target stimulus differs depending on what response is measured [48]. In this study, we found that manual response was unable to capture the atypical attentional profiles toward faces in individuals with WS, whereas eye movements reflected subtle atypical attentional properties. Therefore, we tested the following hypotheses by measuring both manual response and gaze behavior to overcome differences, depending on the effector.

We introduced a visual search paradigm to test two hypotheses regarding the detection of fearful faces in individuals with WS. First, we hypothesized that if a fearful face is a salient visual stimulus to participants compared to a neutral face, then visual search asymmetry would be observed as we found in a preliminary study using the same stimulus set among typically developed adults. This will be characterized by the reaction time for searching for an upright fearful face among neutral faces being shorter than when searching for an upright neutral face among fearful faces for control groups. If this tendency also holds true for individuals with WS, then searching for an upright fearful face among upright neutral faces would be more efficient than searching for an upright neutral face among upright fearful faces. Second, if the processing of fearful faces is dependent on configural processing, then search asymmetry would be observed only in upright faces, but it would be diminished in inverted faces. As the atypicality of inverted face processing in individuals with WS has shown inconsistent findings as mentioned above, we introduced experimental manipulation of the orientation of faces to explore the ability of configural processing of fearful faces.

## Methods

### Participants

Thirteen individuals with WS participated in the experiment (ten males and three females, age range 8; 10–25; 0, mean age 15.7), as shown in Table 1. All participants had previously been phenotypically diagnosed by clinicians, and the diagnosis was subsequently confirmed using fluorescence in situ hybridization analysis. Mental age was measured using the test of Raven’s Colored Progressive Matrices (RCPM) [49, 50].

**Table 1:** Participant information

Group	*N*	Chronological age	
	(F/M)	Mean (Year) Range (years; months)	RCPM score
WS	13 (3/10)	15.7 ± 5.2 (8; 10–25; 0)	18.8 ± 4.7 (13–31)
MA	13 (8/5)	6.0 ± 0.7 (5; 0–7; 4)	20.3 ± 4.4 (13–27)
CA	13 (3/10)	16.0 ± 5.9 (8; 11–27; 11)	N/A

Mean ± SD

A total of 26 typically developed children, adolescents, and adults from nearby elementary schools, junior high schools, high schools, and universities were recruited as control participants (Table 1). For the mentally age-matched (MA) group, 13 children (five males and eight females, age range 5; 0–7; 4, mean age 6.0) were recruited and matched to the WS group based on nonverbal ability as measured by the RCPM. For the chronologically age-matched (CA) group, 13 individuals were recruited and individually matched by age to participants in the WS group (ten males and three females, age range 8; 11–27; 11, mean age 16.0). In terms of the RCPM scores, there were no group differences between the WS and matched MA groups in the first analysis (WS mean 18.8, MA mean 20.3, *p* = 0.23). With regard to the chronological age, there were no significant differences between the WS and CA groups in the second analysis (WS mean 15.7 years, CA mean 16.0 years, *p* = 0.88). All the children, their parents, and the adult participants provided informed consent to take part in the study, which was approved by the ethics committee at the Institute for Developmental Research at the Aichi Human Service Center (Reference Number: 04-08).

### Stimuli and apparatus

The experiment was conducted using a computer (HP Pavilion Desktop, h8-1060jp) with Tobii Studio and E-prime 2.0 software (Psychology Software Tools, Inc., PA, USA), as well as the E-prime extension for Tobii (Tobii, Inc., Stockholm, Sweden). Stimuli were presented on a 24-in. LCD color monitor (Iiyama, PLE2407HDS), placed approximately 60 cm from the observer.

**Fig. 1:**
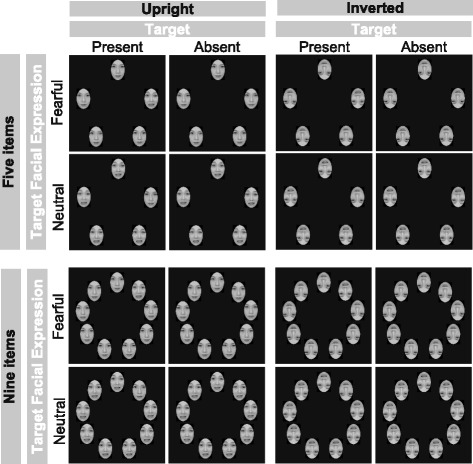
All experimental conditions. Examples of all experimental conditions were displayed (including both five- and nine-item conditions)

Both neutral and fearful faces were taken from the ATR database (Kyoyo, Japan). Five or nine faces were displayed in a circular configuration (see Fig. 1). The faces were various grayscale images sized to fit within a 6.3° × 4.2° square. The averaged luminance for all objects was equated using the SHINE toolbox [51]. The center of each object was located at approximately 11° from the center of the display.

### Design and procedures

In the experiment, we distinguished four factors: target facial expression (fearful vs. neutral), orientation (upright vs. inverted), array size (five vs. nine), and presence of the target (present vs. absent). One of the four possible combinations of the target condition (target facial expression and orientation) served as a block, with a total of four blocks presented over the course of the experiment. Within each block, the vertical orientation of distractors was always the same as that of the targets, with only the number of faces differing (five items or nine items); each array size appeared for an equal number of times. Each block consisted of 36 test trials, preceded by four practice trials. Each block comprised 36 trials of four experimental conditions (nine trials per experimental condition). Accordingly, each participant went through a total of 144 trials for the four experimental conditions. Within each block, the target was present in 50% of trials and was absent in the other 50% (i.e., it was present or absent for 18 trials each). The presentation order of each trial as well as the order of the blocks was randomized across participants. Between sessions, participants were given a 1–2-min break if required. The entire duration of the instruction, practice, calibration, and actual experiment was about 15–20 min. In our preliminary study, we found a search asymmetry effect in nine trials with adults. Task demands were minimized for individuals in both the WS and MA groups. Thus, we have chosen the number of the trials.

Behavioral responses were reported via a custom-made response box with two large buttons. Eye movements were recorded using the Tobii X60 eye-tracking system (Tobii, Inc., Stockholm, Sweden). The eye-tracking system was completely noninvasive, and artificial constraints on head or body movements were not necessary. The system tracked both eyes with an accuracy of 0.5° and a sampling rate of 60 Hz. The eye tracker was calibrated for each participant, using a five-point calibration for each eye.

### Task and procedure

**Fig. 2:**
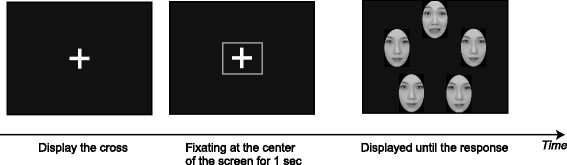
Experimental procedure. The fixation cross was displayed at the center of the screen. If a participant fixates on the fixation cross for 1 s, stimulus array is displayed. After response button is pressed, stimuli disappear

To record reliable eye movement data in each trial for younger children and people with WS, participants were required to attend to a fixation cross at the center of the screen for 1 s to initiate each trial (Fig. 2). If the system detected a 1-s period of fixation at the center of the screen, a stimulus was displayed. By using an eye-tracking system, we could record reliable behavioral and eye movement data for each trial from individuals with WS and typical controls, as visual stimuli were displayed only when participants fixated on the center of the screen for 1 s. Participants were asked to judge as quickly and accurately as possible whether the target facial expression was present in each array and to register their response by pressing one of the two buttons (left-hand side and right-hand side) on the response box. Seven participants were asked to press the left button using their left hand if they found a target facial expression and to press the right button with their right hand if they did not find a target facial expression. The other participants were asked to use their right hand on sighting a target facial expression, being given the opposite instructions. No feedback was given to participants.

### Data analysis

We analyzed both behavioral responses and eye movements. We included only the correct response data, and if the reaction time was above or below the 3 SD from the mean, the trial was excluded from further analysis. For behavioral responses, both reaction time and percent correct were analyzed using a mixed design-repeated measure of analysis of variance (ANOVA). A five-way ANOVA was applied to reaction time and percent correct. The groups (Williams syndrome; WS, mentally age-matched; MA, and chronologically age-matched; CA) were used as between-subject factors and the target facial expression (fearful vs. neutral), orientation (upright vs. inverted), array size (five vs. nine), and presence of the target (present vs. absent) were used as within-subject factors.

For gaze behaviors, we defined areas of interest (AOI) to assess how long it took to reach the initial fixation on the target facial expression and a distractor face. In particular, we were interested in how latencies toward the target facial expression and distractor face were modulated by the type of target facial expression (i.e., fearful or neutral) and orientation depending on the group in the target present condition. One area of interest was assigned for each item in each array; each area was a circle encompassing the whole image. The latency toward the target was defined as the time to get to the AOI of the target face. The latency toward the distractor face was defined as the fastest first fixation on any of the four or eight distractor faces, regardless of their position. As in the behavioral analysis, a five-way ANOVA was applied to the latency of the initial fixation on the target facial expression or distractor face. Groups were used as between-subject factors (WS, MA, and CA). The size array (five vs. nine), orientation (upright vs. inverted), target facial expression (fearful vs. neutral), and target or distractor faces (target face vs. distractor faces) were used as within-subject factors. Tukey’s HSD was applied for multiple comparisons. In the analysis, if the assumption of sphericity was violated in Mauchly’s sphericity test, the Greenhouse–Geisser epsilon coefficient was used to correct the degrees of freedom. Both the *F* and *p* values were then recalculated, and we considered statistical significance to be *p* < 0.05.

## Results

Due to the small number of trials for each condition and relatively large individual variation in the reaction times (RTs), mean RTs were used for the analyses [25, 52]. Note that all participants had at least six valid trials for RT analyses for each condition, as in the previous study [25].

### Reaction times

**Fig. 3:**
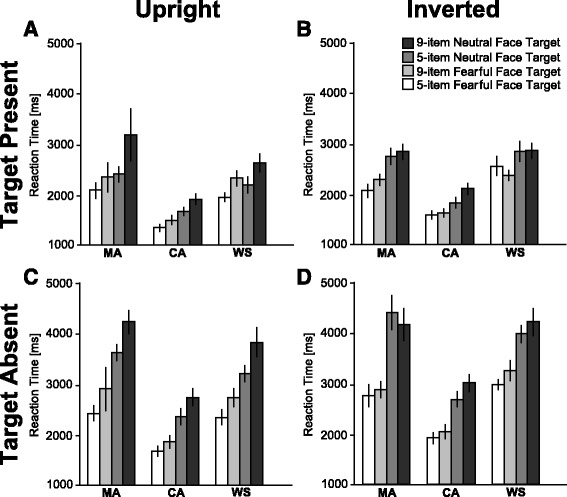
Mean reaction times. **a** *Upright*, target present condition. **b** *Inverted*, target present condition. **c** *Upright*, target absent condition. **d** *Inverted*, target absent condition. Each *color* indicates a combination of target facial expressions and array size, as shown in the figure. *Error bars* indicate standard error of mean (SEM)

For reaction time, as shown in Fig. 3 (a summary of the statistical analysis is shown in Table 2), we found significant main effects, two-way, three-way, and a four-way interaction. Follow-up analyses have shown that we did not find significant group differences in both array sizes although we found group differences in the face orientation and target facial expression condition. However, overall, we did not find group differences in terms of visual search asymmetry; there were prolonged RTs for searching for a neutral face among fearful faces, but these were not obtained for searching for a fearful face among neutral faces.

**Table 2:** The results of statistical analysis for the reaction time

	Degrees of freedom	*F* value	*p* value	*η*_*p*_^2^
Group	2, 36	16.8	0.001**	0.48
Orientation	1, 36	16.7	0.001**	0.32
Orientation × group	2, 36	2.63	0.09	0.13
Target facial expression	1, 36	13.2	0.001**	0.27
Target facial expression × group	2, 36	0.097	0.91	0.005
Array size	1, 36	288.5	0.001**	0.89
Array size × group	2, 36	7.69	0.002**	0.30
Presence of the target	1, 36	340.5	0.001**	0.90
Presence of the target × group	2, 36	6.10	0.005**	0.25
Orientation × target facial expression	1, 36	5.93	0.02*	0.14
Orientation × target facial expression × group	2, 36	1.14	0.33	0.060
Orientation × array size	1, 36	1.75	0.20	0.046
Orientation × array size × group	2, 36	0.21	0.81	0.011
Target facial expression × array size	1, 36	6.34	0.016*	0.15
Target facial expression × array size × group	2, 36	0.85	0.44	0.05
Orientation × target facial expression × array size	1, 36	4.78	0.036*	0.12
*Orientation* × *target facial expression* × *array size* × *group*	*2*, *36*	*5.35*	0.009**	*0.23*
Orientation × presence of the target	1, 36	6.10	0.02*	0.15
Orientation × presence of the target × group	2, 36	0.63	0.54	0.03
Target facial expression × presence of the target	1, 36	0.77	0.39	0.02
Target facial expression × presence of the target × group	2, 36	1.45	0.25	0.05
Orientation × target facial expression × presence of the target	1, 36	0.004	0.95	0.00
Orientation × target facial expression × presence of the target × group	2, 36	2.26	0.12	0.11
Array size × presence of the target	1, 36	100.0	0.001**	0.74
Array size × presence of the target × group	2, 36	2.04	0.15	0.10
Orientation × array size × presence of the target	1, 36	0.11	0.75	0.003
Orientation × array size × presence of the target × group	2, 36	0.32	0.73	0.02
Target facial expression × array size × presence of the target	1, 36	0.68	0.42	0.02
Target facial expression × array size × presence of the target × group	2, 36	0.80	0.46	0.04
Orientation × target facial expression × array size × presence of the target	1, 36	0.39	0.54	0.01
Orientation × target facial expression × array size × presence of the target × group	2, 36	0.63	0.54	0.03

*p < 0.05; **p < 0.01

Italics indicate the significant effect involving the search asymmetry effect across all groups

We firstly report main effects of the analysis. We found significant main effects for group [*p* < 0.01; faster RTs for the CA group than those for the MA and WS groups (*ps* < 0.01) but not significant between the MA and CA groups (WS 2899.7 ms, MA 2967.7 ms, and CA 1996.5 ms)] and target facial expression [*p* < 0.01; faster RTs for fearful face target than those for neutral face target (fearful face target 2491.4 ms, neutral face target 2751.1 ms)]. Further, array size [faster RTs for five-item than those for nine-item (five-item 2246.1 ms, nine-item 2996.5 ms, *p* < 0.01)] and presence of target [*p* < 0.01, faster RTs for target present than those for target absent (target present 2227.0 ms, target absent 3015.6 ms)] were also significant. Moreover, the main effect of orientation was significant [*p* < 0.01; faster RTs for upright than those for inverted (upright 2483.8 ms, inverted 2758.8 ms)].

With regard to interactions, several interactions were significant as shown in Table 2. As we are interested in whether the search asymmetry effect was observed in the WS group and the differential search asymmetry across groups, we firstly focused on significant interactions that were involved in the target facial expression and group. As the four-way interaction of group × orientation × target facial expression × array size was significant, we further explore the nature of the simple main effect.

### Search asymmetry effect across groups

For the simple main effects of group, we found differential search asymmetry effects across groups. For the WS group, the main effects of orientation (*p* < 0.01), target facial expression (*p* < 0.05), and array size (*p* < 0.01) were significant. Furthermore, a two-way interaction of orientation × target facial expression was significant (*p* < 0.05). This suggests that RTs for fearful faces were significantly faster than those for neutral faces only in the upright face condition (*p* < 0.01) but not in the inverted face condition (*p* = 0.47). Furthermore, the RTs for the upright face were significantly faster than those for the inverted face condition in both fearful (*p* < 0.01) and neutral faces (*p* < 0.01). Furthermore, RTs for the nine-item condition were significantly longer than those for the five-item condition (3231.1 vs. 2568.3 ms).

For the MA group, the main effect of array size was significant (*p* < 0.01), suggesting that the RTs for the five-item condition were significantly lower than those for the nine-item condition. The interaction of orientation × target facial expression × array size was significant (*p* < 0.01). This suggests that the RTs for the fearful face condition were significantly faster than those for the neutral face condition in the upright and the nine-item condition (*p* < 0.01). Further, the RTs for the upright face condition were faster than those for the inverted face condition in fearful face detection and the nine-item condition (*p* < 0.01). Furthermore, RTs for the five-item condition were significantly faster than those for the nine-item condition (*Fs* > 47.0, *ps* < 0.01).

For the CA group, the main effects of orientation (*p* < 0.01), target facial expression (*p* < 0.01), and array size (*p* < 0.01) were significant. Furthermore, the interaction of target facial expression × array size was significant (*p* < 0.01). This suggests that the RTs for fearful face were significantly faster than those for neutral face in both five-item (*p* < 0.05) and nine-item (*p* < 0.01) conditions. Further, RTs for the nine-item condition were significantly longer than those for the five-item condition in both fearful (*p* < 0.01) and neutral faces (*p* < 0.01).

### Search asymmetry effect depends on face orientation

For the simple main effects of orientation of faces, the search asymmetry effect was observed for both upright and inverted faces, but the effect was different across groups. Concerning the upright face condition, the main effects of group (*p* < 0.01), target facial expression (*p* < 0.01), and array size (*p* < 0.01) were significant. Furthermore, the two-way interactions of group × array size (*p* < 0.01) and target facial expression × array size (*p* < 0.01) were significant. Regarding the interaction of target facial expression × array size, the RTs for the neutral face target were significantly longer than those for the fearful face target in both array sizes (*Fs* > 6.1, *ps* < 0.02). Further, RTs for the nine-item condition were significantly longer than those for the five-item condition in both the target facial expressions (*Fs* > 152.8, *ps* < 0.01). Furthermore, RTs for the nine-item condition were significantly longer than those for the five-item condition for all groups (*Fs* > 71.0, *ps* < 0.01). Regarding the group differences, the RTs for both the MA and WS groups were significantly longer than those for the CA group (*ps* < 0.05) in both the five- and the nine-item conditions. However, no significant differences were found between the MA and WS groups (*ps* > 0.08).

As for the inverted face condition, the main effects of group (*p* < 0.01) and array size (*p* < 0.01) were significant. Furthermore, the two-way interaction of group × array size (*p* < 0.01) and the three-way interactions of group × target facial expression × array size (*p* < 0.05) were significant. The subsequent simple main effects of the three-way interaction revealed that the RTs for the neutral face were significantly longer than those for the fearful face for the nine-item condition in the CA group. Furthermore, the RTs for the nine-item condition were significantly longer than those for the five-item condition in all groups (*Fs* > 41.2, *ps* < 0.01). Other simple main effects did not reach statistical significance (*Fs* < 2.3, *ps* > 0.13). Regarding group differences, the RTs for both the MA and WS groups were significantly longer than those for the CA group (*ps* < 0.01) in both five- and nine-item conditions. However, no significant differences between the MA and WS groups were observed (*ps* > 0.10).

### Search asymmetry effect depends on the size of items

For the simple main effects of size, the search asymmetry effect was observed only for the nine-item condition but not for the five-item condition. For the five-item condition, the main effects of the group (*p* < 0.01), orientation (*p* < 0.01), and target facial expression (*p* < 0.01) were significant.

For the nine-item condition, the main effects of group (*p* < 0.01), orientation (*p* < 0.01), and target facial expression (*p* < 0.01) were significant. Furthermore, the two-way interaction of orientation × target facial expression was significant (*p* < 0.01). This suggests that the RTs for fearful face were significantly faster than those for neutral face only in the upright condition (*p* < 0.01). Further, the RTs for the upright face were significantly faster than those for the inverted face in the fearful face condition (*p* < 0.01).

### Group differences across facial expressions

For the simple main effects of target facial expressions, the group differences were observed for both upright and inverted faces. For the fearful target facial expression condition, the main effects of group (*p* < 0.01), orientation (*p* < 0.01), and array size (*p* < 0.01) were significant. Furthermore, the two-way interactions of group × orientation (*p* < 0.01), group × array size (*p* < 0.01), and orientation × array size (*p* < 0.05) were significant. This suggests that the RTs for upright faces were significantly shorter than those for inverted faces in all groups (*Fs* > 5.4, *ps* < 0.05). Furthermore, the RTs for the nine-item condition were significantly longer than those for the five-item condition in all groups (*Fs* > 56.8, *ps* < 0.01). Regarding group differences, the RTs for both the MA and WS groups were significantly longer than those for the CA group (*ps* < 0.01) in both the five- and nine-item as well as both upright and inverted conditions. However, no significant differences were observed between the MA and WS groups (*ps* > 0.17).

For the neutral target facial expression condition, the main effects of group (*p* < 0.01) and array size (*p* < 0.01) were significant. Furthermore, the two-way interactions of group × array size were significant (*p* < 0.05). It suggests that the RTs for the nine-item condition were significantly longer than those for the five-item condition (*Fs* > 47.2, *ps* < 0.01). Regarding the group differences, the RTs for both MA and WS groups were significantly longer than those for the CA group (*ps* < 0.01). However, no significant differences were observed between the MA and WS groups (*ps* > 0.37).

### Accuracy

**Fig. 4:**
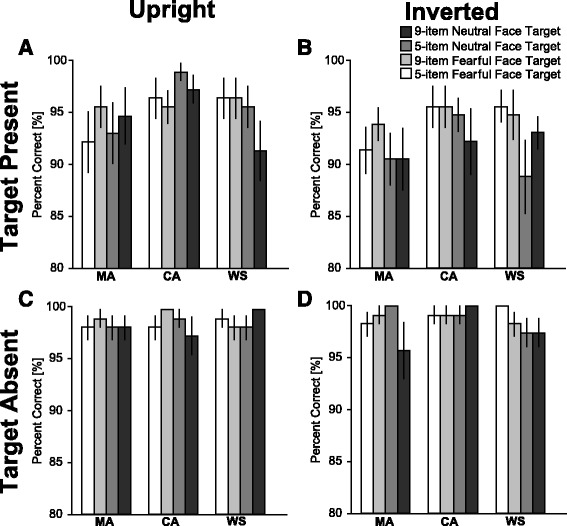
Mean percent correct. **a** *Upright*, target present condition. **b** *Inverted*, target present condition. **c** *Upright*, target absent condition. **d** *Inverted*, target absent condition. Each *color* indicates a combination of target facial expression and array size as shown in the figure. *Error bars* indicate SEM

For performance accuracy (Fig. 4; a summary of the statistical analysis is shown in Table 3), we found significant main effects in the array size (*p* < 0.01), orientation (*p* < 0.05), and the presence of the target (*p* < 0.01). In addition, we found significant interactions of orientation × presence of the target (*p* < 0.05) and orientation × array size (*p* < 0.05). However, other effects did not reach statistical significance (*Fs* < 1.2, *ps* > 0.27).

**Table 3:** The results of statistical analysis for the accuracy

	Degrees of freedom	*F* value	*p* value	*η*_*p*_^2^
Group	2, 36	1.59	0.22	0.08
Orientation	1, 36	6.13	0.018*	0.15
Orientation × group	2, 36	0.51	0.95	0.003
Target facial expression	1, 36	0.007	0.93	0.000
Target facial expression × group	2, 36	0.67	0.52	0.04
Array size	1, 36	8.15	0.007**	0.19
Array size × group	2, 36	2.69	0.08	0.13
Presence of the target	1, 36	43.3	0.001**	0.55
Presence of the target × group	2, 36	1.14	0.33	0.60
Orientation × target facial expression	1, 36	0.005	0.94	0.00
Orientation × target facial expression × group	2, 36	0.74	0.48	0.04
Orientation × array size	1, 36	4.26	0.046*	0.12
Orientation × array size × group	2, 36	0.029	0.97	0.002
Target facial expression × array size	1, 36	0.76	0.39	0.02
Target facial expression × array size × group	2, 36	1.16	0.32	0.06
Orientation × target facial expression × array size	1, 36	0.31	0.58	0.02
Orientation × target facial expression × array size × group	2, 36	1.21	0.31	0.06
Orientation × presence of the target	1, 36	4.45	0.042*	0.11
Orientation × presence of the target × group	2, 36	0.28	0.76	0.03
Target facial expression × presence of the target	1, 36	0.11	0.74	0.003
Target facial expression × presence of the target × group	2, 36	1.23	0.31	0.06
Orientation × target facial expression × presence of the target	1, 36	0.95	0.34	0.03
Orientation × target facial expression × presence of the target × group	2, 36	0.79	0.46	0.04
Array size × presence of the target	1, 36	1.24	0.27	0.33
Array size × presence of the target × group	2, 36	1.04	0.36	0.06
Orientation × array size × presence of the target	1, 36	1.52	0.23	0.04
Orientation × array size × presence of the target × group	2, 36	1.16	0.33	0.06
Target facial expression × array size × presence of the target	1, 36	0.05	0.83	0.001
Target facial expression × array size × presence of the target × group	2, 36	0.15	0.86	0.01
Orientation × target facial expression × array size × presence of the target	1, 36	0.42	0.52	0.01
Orientation × target facial expression × array size × presence of the target × group	2, 36	1.41	0.26	0.07

*p < 0.05; **p < 0.01

To explore the nature of the interaction of orientation × presence of the target, tests of the simple main effect were performed. The simple main effect of orientation was significant within the target present condition (*p* < 0.01) but not within the target absent condition (*p* = 0.92). This suggests that the accuracies for the upright condition were significantly higher than those for the inverted condition when the target was present (95.4 vs. 93.1%).

Further, to explore the nature of the interaction of orientation × array size, the simple main effect of orientation was significant in the nine-item condition (*p* < 0.01) but not in the five-item condition (*p* = 0.46). This suggests that the accuracies for the upright condition were significantly better than those in the inverted condition in the nine-item array (96.9 vs. 95.0%). Further, the simple main effect of the size of the array was significant in the inverted condition (*p* < 0.01) but not in the upright condition (*p* = 0.58). This suggests that the accuracies for the five-item condition were significantly better for the nine-item condition when the faces were upside down (96.8 vs. 95.0%).

### Gaze behavior (initial saccade latency toward the target or distractor)

As we fully recorded fixation duration data from 10 individuals with WS (one was unable to record complete eye movement data in all experimental conditions due to a technical problem, and two were excluded as the individuals did not fixate the predefined area of interest in some experimental conditions) and 12 individuals among MA and CA participants (individual data for one MA were missing due to a technical problem, and data from one CA individual did not show fixation on the predefined area of interest in some experimental conditions).

**Fig. 5:**
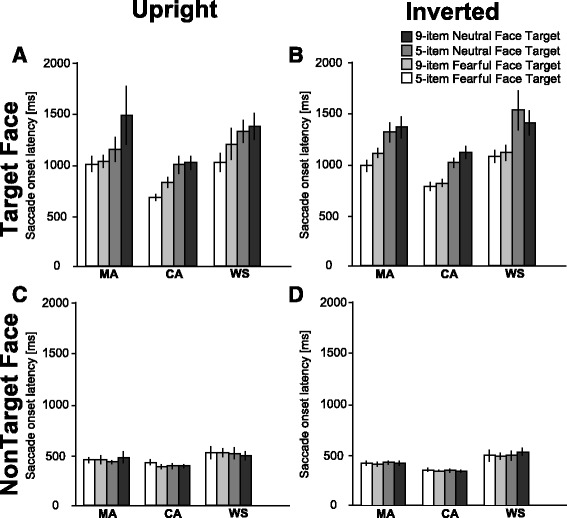
Saccade onset latency toward a target or a distractor face for the condition where the target is present (target present condition). **a** *Upright*, target present condition. **b** *Inverted*, target present condition. **c** *Upright*, target absent condition. **d** *Inverted*, target absent condition. Each *color* indicates a combination of target facial expression and array size as shown in the figure. *Error bars* indicate SEM

We further analyzed the latencies of initial fixation on target facial expression or distractor faces in the target present condition (Fig. 5; a summary of the statistical analysis is shown in Table 4). We first report the main effects of the analysis. We found significant main effects for the array size (*p* < 0.01), group (*p* < 0.01), and target or distractor faces (*p* < 0.01). These results indicate that the latencies for the five-item condition were significantly faster than those for the nine-item condition (669.4 vs. 843.2 ms), and the latencies for the distractor faces were significantly faster than those for the target facial expression (433.3 vs. 1109.3 ms). Further, the latencies for both the WS and MA groups were significantly slower than those for the CA group (*ps* < 0.01; WS 884.0 ms, MA 809.7 ms, CA 639.0 ms).

**Table 4:** The results of statistical analysis for the gaze behavior

	Degrees of freedom	*F* value	*p* value	*η*_*p*_^2^
Group	2, 31	11.0	0.001**	0.42
Orientation	1, 31	0.089	0.77	0.003
Orientation × group	2, 31	0.13	0.88	0.008
Target facial expression	1, 31	2.82	0.10	0.08
Target facial expression × group	2, 31	0.46	0.63	0.03
Array size	1, 31	40.9	0.001**	0.57
Array size × group	2, 31	0.14	0.87	0.009
Target or distractor faces	1, 31	628.9	0.001**	0.95
Target or distractor faces × group	2, 31	7.17	0.001**	0.32
Orientation × target facial expression	1, 31	1.00	0.33	0.03
Orientation × target facial expression × group	2, 31	0.26	0.77	0.02
Orientation × array size	1, 31	0.60	0.44	0.02
Orientation × array size × group	2, 31	0.46	0.64	0.03
Target facial expression × array size	1, 31	0.002	0.97	0.00
Target facial expression × array size × group	2, 31	0.88	0.42	0.05
Orientation × target facial expression × array size	1, 31	0.30	0.59	0.01
Orientation × target facial expression × array size × group	2, 31	2.17	0.13	0.12
Orientation × target or distractor faces	1, 31	3.38	0.08	0.10
Orientation × target or distractor faces × group	2, 31	0.20	0.82	0.01
*Target facial expression* × *target or distractor faces*	*1*, *31*	*4.39*	0.045*	*0.12*
Target facial expression × target or distractor faces × group	2, 31	0.41	0.67	0.03
Orientation × target facial expression × target or distractor faces	1, 31	1.88	0.18	0.06
Orientation × target facial expression × target or distractor faces × group	2, 31	0.50	0.61	0.03
Array size × target or distractor faces	1, 31	47.1	0.001**	0.60
Array size × target or distractor faces × group	2, 31	0.04	0.97	0.002
Orientation × array size × target or distractor faces	1, 31	0.13	0.73	0.004
Orientation × array size × target or distractor faces × group	2, 31	0.19	0.81	0.01
Target facial expression × array size × target or distractor faces	1, 31	0.26	0.62	0.008
Target facial expression × array size × target or distractor faces × group	2, 31	0.92	0.41	0.06
Orientation × target facial expression × array size × target or distractor faces	1, 31	0.23	0.63	0.01
Orientation × target facial expression × array size × target or distractor faces × group	2, 31	1.75	0.19	0.10

**p < 0.01; *p < 0.05

Italics indicate the significant effect involving the search asymmetry effect across all groups

### Search asymmetry effect across groups

As we are interested in whether the search asymmetry effect was observed in the WS group, we first focused on significant interactions that were involved in the target facial expression and group. With regard to the interaction, we found significant interactions of target facial expression × target or distractor faces (*p* < 0.05), group × target or distractor faces (*p* < 0.01), and array size × target (*p* < 0.01).

To explore the nature of the interaction of target facial expression × target or distractor faces, tests of the simple main effect were performed. The simple main effect of the target facial expression was significant within the target face condition (*p* < 0.01) but not within the distractor faces (*p* = 0.94). This suggests that the latency of the neutral target facial expression was significantly longer than that of the fearful target facial expression in all groups. For both the target facial expression conditions, the latency of the target face was significantly longer than that of the distractor faces in both the fearful (*p* < 0.01) and neutral (*p* < 0.01) target face conditions.

### The effect of group depends on the presence of target

To explore the nature of the interaction of group × target or distractor faces, tests of the simple main effect were performed. The simple main effects of the target or distractor faces were significant within all groups (*ps* < 0.01). This suggests that the latencies toward the target facial expression were significantly longer than those toward the distractor faces in all groups. The main effect of the group was significant within the target face (*p* < 0.01) but not significant within the distractor faces (*p* = 0.10). This suggests that the latencies in both the WS and MA groups were significantly longer than those in the CA group toward a target face (*ps* < 0.01), but the latencies between the WS and MA groups were not significant (*p* = 0.18).

### The effect of the item size depends on the presence of the target

We further explored the nature of the interaction of the array size × target or distractor faces. The simple main effect of the target face was significant within the five-item (*p* < 0.01) and the nine-item (*p* < 0.01) conditions. This suggests that the latencies toward the distractor faces were significantly faster than those toward the target face in both array size conditions. Further, the simple main effect of the array size was significant within the target face (*p* < 0.01) but not the distractor faces (*p* = 0.95). This indicates that the latencies for the nine-item condition were significantly longer than those for the five-item condition toward a target face.

## Discussion

The current study was designed to test whether the detection mechanisms for fearful faces are preserved in individuals with WS by introducing a visual search paradigm. We assessed search asymmetry as an index by measuring both manual responses and gaze behaviors. Search asymmetry was defined to occur when a neutral face was displayed as a target facial expression among fearful faces; RT was longer compared to when a fearful face was the target among neutral faces. In line with previous behavioral studies on visual search study for emotional faces [28, 30], search asymmetry was also found between fearful and neutral faces.

Overall, although reaction time was prolonged in both the WS and MA groups compared to the CA group, we did not find atypical search asymmetries in the WS group. This suggests that the cognitive mechanisms of detecting a fearful face can be preserved in the WS group when compared to the control groups. However, the effect of search asymmetry was slightly different in each group. For the WS group, we found a significant interaction of orientation × target facial expression, suggesting that the RT for neutral face detection was longer than that for fearful face detection only in the upright face condition, but this effect was diminished in the inverted face condition. It suggests that the search asymmetry holds only when the configural processing is preserved in the WS group. For the MA group, we found a significant interaction of orientation × target facial expression × size array, suggesting that search asymmetry was found only in the nine-item condition, not in the five-item condition for upright faces, and was not found in the inverted faces. This suggests that search asymmetry was prominent when the task was difficult. For the CA group, contrary to both the WS and MA groups, search asymmetry was found in both the upright and inverted conditions, irrespective of the array size. This suggests that local features of faces were used in the task.

In light of our initial hypotheses, if the amygdala account were true for explaining hypersociability observed in individuals with WS, we should expect search asymmetry to be diminished in individuals with WS. However, we observed search asymmetry in individuals with WS, suggesting that a fearful face is more salient than a neutral face. In light of the two accounts regarding hypersociability in individuals with WS, it is possible that the amygdala account does not fully fit current findings. One of the plausible reasons why we could not find the atypicality of the search asymmetry would be due to the task differences contrasting with previous studies. Most previous studies have introduced face “perception”/“recognition” tasks [53] or matching tasks [19] and have shown reduced neural activities in the amygdala region. In contrast with these experimental paradigms, the current visual search task we used might not capture the distinctive aspects of processing social affective information shown by individuals with WS.

The differential experimental paradigm might tap differential functional aspects of amygdala processing. A previous neuropsychological study has demonstrated that an individual with complete bilateral amygdala lesions who cannot recognize fear in faces nonetheless showed normal rapid detection and nonconscious processing of those same fearful faces [20]. Other evidence has suggested that individuals with amygdala lesions detect emotional targets more efficiently than neutral targets when compared with healthy controls [54]. It is likely that the amygdala is not necessary for emotion-guided visual search or is not essential for the early stage of fear processing. Rather, the amygdala may modulate later cognitive processes such as recognition and social judgment [20]. If this view is true, it is possible that the ability to detect fearful faces to be preserved in individuals with WS even though several studies have demonstrated an atypical structural and functional neural activity of the amygdala in individuals with WS [17, 19]. As the currently proposed amygdala theory does not fully distinguish atypicality between “detection” and “recognition” of fearful faces in individuals with WS, further studies should refine the amygdala account to explain which aspect of fear-related processing is atypical and lead to hypersociability in individuals with WS.

Because only fearful and neutral expressions were used in the current experiment, it is possible that the search asymmetries found may be related to distinguishing between (and detecting faster) emotional and neutral facial expressions. Therefore, it would not be specific to fear detection as a signal of threat. To verify this point, it is worthwhile to introduce the other types of facial expression as control. By introducing happy, fearful, and neutral facial expressions, Haas and colleagues [53] have shown the amygdala reactivity to happy faces and absent or attenuated amygdala reactivity to fearful facial expressions. Moreover, the abnormal amygdala reactivity in WS might possibly function to increase attention to and encode happy facial expressions and decrease arousal to fearful expressions. As we did not include positive facial expressions such as happy faces in our current task, we cannot exclude the possibility of the atypical amygdala function in individuals with WS. As the increased amygdala responses to happy facial expressions in individuals with WS, it might represent several psychological processes linked to the amygdala, including attention, arousal, and anxiety. For example, the central nucleus of the amygdala has been strongly linked with attention [55, 56]. Therefore, it would be beneficial to test whether the search asymmetry effect would be observed between happy and neutral faces as compared to fearful and neutral faces as the ones found in the current experiment.

Because we have introduced the visual search paradigm using both fearful and neutral faces to tap the function of the amygdala, we cannot directly test whether the alternative frontal lobe theory can account for the hypersociability reported in individuals with WS. However, we found that, overall, the reaction time was significantly longer than that for other control groups and the reaction time was prolonged as the set size increases in comparison with control groups. As the task difficulty of the visual search modulates the neural activities in the bilateral ventrolateral prefrontal cortex and right dorsolateral prefrontal cortex [57], it seems that the functioning of the prefrontal cortex was not atypical in individuals with WS in the current experiment.

When faces were presented upside down, we found that search asymmetry was diminished in the WS group, contrasting with the performances in the CA group. This suggests that search asymmetry was not induced by the local elements of faces but by the processing of global configuration of faces. This view contrasts somewhat with previous findings that fail to obtain evidence for an inversion effect in individuals with WS [31–33]. Studies have also investigated this phenomenon using event-related potentials [45] and evoked fields [46]. These discrepancies may be explicable from task differences as the current task requires the identification of an emotional expression that seems to be preserved [7, 8].

As in our previous study [47, 48], both coarse measures such as manual responses and fine measures such as eye movement were simultaneously recorded during visual search. In our previous experiment, eye movements reflected subtle atypical attentional properties; however, manual responses were unable to capture atypical attentional profiles toward upright faces in individuals with WS. Therefore, we measured both manual responses and gaze behaviors during the visual search task and found the effects of search asymmetry. Most previous studies have used a single modality, such as manual responses or eye movements, for measuring the attentional process in individuals with WS. We have further shown that both measures reflect search asymmetry. We believe that the measurements of both manual and eye movements are useful to validate the effect.

Although our current study provides new insights into the mechanisms underlying the detection of negative social stimuli in WS, there are several limitations. First, it is possible that the visual search task used does not capture the distinctive aspects of processing social affective information shown by individuals with WS. Because only fearful and neutral expressions were used in the experiment, it is possible that the search asymmetries found may be related to distinguishing between emotional facial expressions and neutral facial expressions. Therefore, it may not be specific to detection of fear as a signal of threat. Second, the number of participants was rather small for tracing developmental changes in individuals with WS. Further studies are needed to address the developmental changes in the performances of the search asymmetry. Third, we only analyzed (a minimum of) nine trials, as we introduced many experimental conditions in our current experiment. We think that further validation is needed to ascertain whether the effect will be observed.

## Conclusions

In conclusion, we did not find any atypical visual search asymmetries in the search for fearful faces in measures of both manual response and eye movement in individuals with WS during a visual search task. This suggests that fearful faces were also salient stimuli compared to neutral faces in people with WS. Our current finding seems to contrast with previous neuroimaging findings regarding the atypical neural activities related to fearful face processing in individuals with WS. However, this finding can give a clue to formulate the atypical fear-related processing, such as differential processing, which could be involved in “detecting” and “recognizing” the processing of fearful faces in individuals with WS. We believe that our current findings will contribute to refining theoretical models to explain hypersociability in individuals with WS, particularly the amygdala account, from the viewpoint of conscious and nonconscious processing of fear-related information.

## Abbreviations

ANOVA: Analysis of variance
AOI: Area of interest
CA: Chronological age-matched
MA: Mental age-matched
RCPM: Raven’s Colored Progressive Matrices test
WS: Williams syndrome
